# Detection and population genetic analysis of *kdr* L1014F variant in eastern Ethiopian *Anopheles stephensi*

**DOI:** 10.1016/j.meegid.2022.105235

**Published:** 2022-02-02

**Authors:** Jeanne N. Samake, Solomon Yared, Dejene Getachew, Peter Mumba, Dereje Dengela, Gedeon Yohannes, Sheleme Chibsa, Sae Hee Choi, Joseph Spear, Seth R. Irish, Sarah Zohdy, Meshesha Balkew, Tamar E. Carter

**Affiliations:** a Department of Biology, Baylor University, Waco, TX, USA; b Department of Biology, Jigjiga University, Jigjiga, Ethiopia; c Department of Biology, Dire Dawa University, Dire Dawa, Ethiopia; d PMI VectorLink Project, Abt Associates, Addis Ababa, Ethiopia; e PMI VectorLink Project, Abt Associates, Rockville, MD, USA; f PMI U.S. Agency for International Development (USAID), Addis Ababa, Ethiopia; g U.S. President’s Malaria Initiative, U.S. Centers for Disease Control and Prevention (CDC), Atlanta, GA, USA

**Keywords:** Malaria, Invasive mosquito, *An. Stephensi*, Insecticide resistance, *Kdr* mutation, L1014F

## Abstract

*Anopheles stephensi* is a malaria vector that has been recently introduced into East Africa, where it threatens to increase malaria disease burden. The use of insecticides, especially pyrethroids, is still one of the primary malaria vector control strategies worldwide. The knockdown resistance (*kdr*) mutation in the IIS6 transmembrane segment of the voltage-gated sodium channel (*vgsc*) is one of the main molecular mechanisms of pyrethroid resistance in *Anopheles*. Extensive pyrethroid resistance in *An. stephensi* has been previously reported in Ethiopia. Thus, it is important to determine whether or not the *kdr* mutation is present in *An. stephensi* populations in Ethiopia to inform vector control strategies. In the present study, the *kdr* locus was analyzed in *An. stephensi* collected from ten urban sites (Awash Sebat Kilo, Bati, Dire Dawa, Degehabur, Erer Gota, Godey, Gewane, Jigjiga, Semera, and Kebridehar) situated in Somali, Afar, and Amhara regions, and Dire Dawa Administrative City, to evaluate the frequency and evolution of *kdr* mutations and the association of the mutation with permethrin resistance phenotypes. Permethrin is one of the pyrethroid insecticides used for vector control in eastern Ethiopia. DNA extractions were performed on adult mosquitoes from CDC light trap collections and those raised from larval and pupal collections. PCR and targeted sequencing were used to analyze the IIS6 transmembrane segment of the *vgsc* gene. Of 159 *An. stephensi* specimens analyzed from the population survey, nine (5.7%) carried the *kdr* mutation (L1014F). *An. stephensi* with *kdr* mutations were only observed from Bati, Degehabur, Dire Dawa, Gewane, and Semera. We further selected randomly twenty resistant and twenty susceptible *An. stephensi* mosquitoes from Dire Dawa post-exposure to permethrin and investigated the role of *kdr* in pyrethroid resistance by comparing the *vgsc* gene in the two populations. We found no *kdr* mutations in the permethrin-resistant mosquitoes. Population genetic analysis of the sequences, including neighboring introns, revealed limited evidence of non-neutral evolution (e.g., selection) at this locus. The low *kdr* mutation frequency detected and the lack of *kdr* mutation in the permethrin-resistant mosquitoes suggest the existence of other molecular mechanisms of pyrethroid resistance in eastern Ethiopian *An. stephensi*.

## 1. Introduction

Malaria remains a significant public health threat, with 229 million cases reported worldwide and over 400,000 deaths, mainly in Africa, in 2019 (World Health Organization [WHO], 2020). Thus, the recent detection of the South Asian malaria vector *Anopheles stephensi* in the Horn of Africa has raised concerns about the growing impact on malaria transmission and disease burden (Carter et al., 2018; Faulde et al., 2014; WHO, 2019a). The detection of *An. stephensi* in Djibouti since 2012 has coincided with an exponential increase in malaria cases from 1684 cases in 2013 to 49,402 cases in 2019 (WHO, 2020). Global efforts to control and eliminate malaria led to a significant reduction in malaria case incidence, with a 27% decrease between 2000 and 2015 worldwide (WHO, 2020). This success was mainly due to the use of insecticide-treated nets (ITNs), long-lasting insecticide nets (LLINs), and indoor residual sprayings (IRS) of insecticides (Bhatt et al., 2015). However, the emergence of a new malaria vector in the Horn of Africa and the spread of insecticide resistance in malaria vectors globally threaten malaria control and elimination efforts (Sinka et al., 2020; WHO, 2019a).

One major force driving the spread of insecticide resistance in mosquito populations is the extensive use of insecticides for both public health and agriculture purposes during the past decade (WHO, 2012, 2019b). Pyrethroid resistance in malaria vectors is a significant risk because pyrethroids are among insecticide classes recommended for ITNs and LLINs impregnation (Hougard et al., 2003; WHO, 2019b). One of the molecular mechanisms of pyrethroid resistance in *Anopheles* is target-site insensitivity of sodium channels (Donnelly et al., 2016; Liu et al., 2006; Ranson et al., 2011). Pyrethroids exert their toxic effect by hindering the voltage-gated sodium channels on the mosquito’s neurons, which generally produces fast knockdown properties (David et al., 2013). Mutations in the IIS6 transmembrane segment of the voltage-gated sodium channel (*vgsc*) gene result in target-site resistance known as knockdown resistance (*kdr*), which confers pyrethroid resistance to mosquitoes (Chen et al., 2010; Scott et al., 2015; Silva et al., 2014). Two of the most prevalent *kdr* mutations in *Anopheles* are point mutations that lead to nonsynonymous substitution at amino acid position 1014 in the IIS6 transmembrane of the *vgsc* gene (Silva et al., 2014). The first is the substitution of the leucine amino acid at position 1014 of the sodium channel in the wild-type with the phenylalanine (L1014F) called *kdr-west (kdr-w)* due to its first detection in *Anopheles gambiae* in West Africa (Martinez-Torres et al., 1998; Pinto et al., 2007). The second *kdr* mutation is the substitution of the leucine with the serine amino acid (L1014S), which was first observed in East Africa thus referred to as *kdr-east* (*kdr-e*) (Pinto et al., 2007; Ranson et al., 2000). Since their first detection, both L1014F and L1014S mutations have been reported in West Africa, Central Africa, and East Africa (Djègbè et al., 2018; Lynd et al., 2018; Ondeto et al., 2017). Similarly, *kdr* mutations L1014F and L1014S have been observed in *An. stephensi* in Dubai, Afghanistan, and India (Ahmad et al., 2016; Dykes et al., 2016; Enayati et al., 2003; Gayathri and Murthy, 2006; Singh et al., 2011).

Recent insecticide resistance surveillance in Ethiopia revealed pyrethroid resistance in the *An. stephensi* populations (Balkew et al., 2021; PMI, 2020; Yared et al., 2020). However, the knockdown resistance (*kdr*) mutation in the IIS6 transmembrane segment of the *vgsc* gene was not detected in *An. stephensi* collected in 2016 from Kebridehar, the city where *An. stephensi* was first identified in eastern Ethiopia (Yared et al., 2020). Further investigation of the neighboring intron in these *An. stephensi* revealed variation, which was uncharacteristic of established Culicidae that carry the *kdr* mutation at a high frequency, raising the question about the role of *kdr* in resistance in *An. stephensi* (Carter et al., 2022). Although these studies provide important preliminary insight, more information is needed across eastern Ethiopia to better understand the molecular mechanisms of pyrethroid resistance in *An. stephensi* and to determine whether or not the *kdr* mutation is important in this species.

Here, we first analyzed the *vgsc* gene of *An. stephensi* from eastern Ethiopia to detect the presence or absence of *kdr-west* or *kdr-east* and determine the frequency of *kdr* mutations. Second, we evaluated the neighboring *kdr* introns in the *vgsc* gene to characterize the evolutionary history of the *kdr* mutation in *An. stephensi* from eastern Ethiopia. Additionally, to understand the importance of *kdr* mutation in pyrethroid resistance in *An. stephensi* from eastern Ethiopia, we randomly selected and analyzed the *kdr* locus in phenotypic susceptible and resistant *An. stephensi* specimens from WHO bioassay tests.

## 2. Methods

### 2.1. Sample descriptions

DNA samples analyzed in this study were extracted from 159 (101 lab-reared and 58 wild-caught) adult *An. stephensi* mosquitoes collected in a previous study (Balkew et al., 2020). *An. stephensi* surveys were conducted from August to November 2018 in ten selected sites (Awash Sebat Kilo, Bati, Dire Dawa, Degehabur, Erer Gota, Godey, Gewane, Jigjiga, Kebridehar, Semera) in eastern Ethiopia, particularly in the Somali region, Afar, Amhara region, and Dire Dawa city. Adult mosquitoes were collected using pyrethrum spray sheet collections (PSC) and Centers for Disease Control (CDC) light traps. Larvae and pupae of *Anopheles* were collected from different larval breeding habitats, including artificial water containers, to reduce sampling siblings and reared to adults in field insectaries. After morphological identifications to species, adult *An. stephensi* were tested against insecticides using standard protocol of WHO (2016) as detailed in Balkew et al. (2021). Dead and alive mosquitoes were preserved on silica gel and all specimens were molecularly identified as *An. stephensi*. Detailed collection site descriptions are reported in Balkew et al. (2020). In this study, collection sites were futher categorized (northern, central, and southern) based on proximity to specific major roads and relative location across sites. “Northern” sites included the northern-most sites (Bati, Gewane, Semera) off the B11 and A1 roads (i.e., the road from Mille town to Kombolcha town and the main road between Addis Ababa and Djibouti, respectively). “Central” sites (Awash Sebat Kilo, Dire Dawa, Erer Gota, Jigjiga) were located in the center relative to the rest of the collection sites along the A10 road (i.e., the road between Addis Ababa and Degehabur) running west/east. “Southern” sites were the southern-most sites (Degehabur, Godey, Kebridehar) off the A10 road running north/south.

### 2.2. Amplification and sequencing of kdr loci

**Fig. 1.**
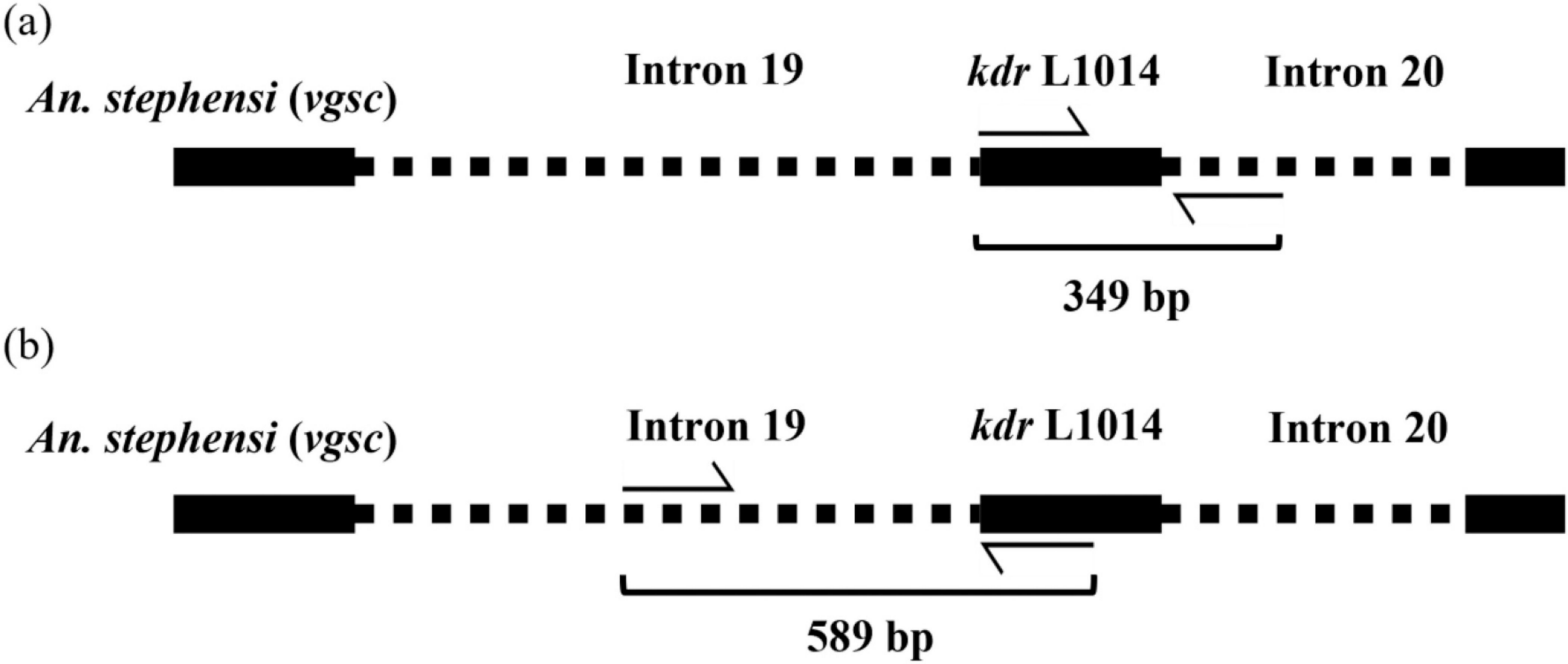
Portions of the *An. stephensi vgsc* gene amplified.

A portion of the *vgsc* gene containing the *kdr* locus and downstream intron 20 were amplified in 159 *An. stephensi* samples using a previously reported polymerase chain reaction (PCR) protocol in Singh et al. (2011) with modifications as detailed in Yared et al. (2020) (Fig. 1, Table 1). Thermal cycling protocol was as follows: 95 °C for 5 min, followed by 35 cycles of 95 °C for 30 s, 50 °C for 30 s, 72 °C for 45 s, and a final extension of 72 °C for 7 min. To further analyze the evolutionary history of the *kdr* mutations in the region, a portion of the adjacent upstream intron (i.e., intron 19) was also amplified using primers designed from the reference sequence ASTE016412 from VectorBase with Primer3 software (Koressaar and Remm, 2007; Untergasser et al., 2012) (Fig. 1, Table 1). Final reagent concentrations and components were 0.5 μM for each primer, 1× Promega GoTAQ HotStart master mix (Promega, Madison, Wisconsin), and water for a total reaction volume of 25ul. The thermal cycling protocol was performed as follows: 94 °C for 5 min, 34 cycles of 94 °C for 40 s, 56 °C for 1 min, 72 °C for 3 min, and a final extension of 72 °C for 10 min. The PCR products were sent to the University of Texas at Austin Center for Biomedical Research Support for sequencing. All amplicons were cleaned using Sera-Mag Speedbead Magnetic Carboxylate Modified Particles (Cytiva, Marlborough, MA) and sequenced using Sanger technology with BigDye chemistry diluted with 5× Sequencing Buffer (Teknova T1099) (EdgeBio, San Jose, CA) and run on an ABI 3730 Genetic Analyzer with POP7 polymer (Thermo Fisher, Santa Clara, CA).

### 2.3. Sequence analysis

DNA sequences were submitted as queries to the National Center for Biotechnology Information’s (NCBI) Basic Local Alignment Search Tool (BLAST) to confirm correct loci were amplified. Sequences were then aligned to identify kdr mutations. The kdr allele and genotype frequencies were then calculated.

We determined the level of diversity in both neighboring introns, upstream and downstream (i.e., intron 19 and intron 20) of the kdr 1014 mutation for additional evidence of selection on that locus. We calculated the number of segregating sites (S), nucleotide diversity (Pi), the estimated number of haplotypes (h), and haplotype diversity (Hd) (i.e., gene diversity) using the program DNAsp v5 (Rozas et al., 2003). Haplotypes were reconstructed using the Phase 2.1 algorithm in DNAsp (Stephens et al., 2001) and confirmed through cloning using NEB PCR cloning kit (New England Biolabs, Ipswich, MA). Adjacent introns, upstream and downstream (i.e., intron 19 and intron 20), were also examined for neutrality using Tajima’s D, Fu’s F, and Fu and Li’s D* and F* tests (Fu et al., 1997; Fu and Li, 1993; Tajima, 1989).

### 2.4. Phylogenetic and network analysis

Phylogenetic analysis was performed with the Ethiopian *An. stephensi kdr* sequences generated in this study and *An. stephensi kdr* sequences retrieved from NCBI. The global *An. stephensi kdr* sequences included three from India (Genbank accession number: JF304952.1, JF304954.1, JF304955.1) (Singh et al., 2011), two from Iran (Genbank accession number: KJ636080.1, KJ676661.1), and one from Sri Lanka (Genbank accession number: MK248685.1) (Surendran et al., 2020). Phylogenetic relationships between the Ethiopian and Genbank sequences were inferred using a maximum likelihood approach with RAxML (Stamatakis, 2014). We applied the GTRGAMMA option that uses the general time reversible (GTR) model of nucleotide substitution with the gamma model of heterogeneity rate (Stamatakis, 2014; Tavaré, 1986). A total of 1000 runs were completed with a strategy to identify the heuristically-best-scoring tree under the maximum likelihood criterion and rapid bootstrap analysis in one run. The tree was rooted at the midpoint. RAxML output was viewed in FigTree, and a final phylogenetic tree was created. Haplotypes network and map were created using the minimum spanning network method in PopArt (Leigh and Bryant, 2015).

### 2.5. Association with permethrin resistance phenotype

To further analyze phenotypic and genotypic associations in observed pyrethroid-resistant *An. stephensi,* we conducted separately, on susceptible and resistant mosquitoes, *kdr* genotyping as described above. The bioassay was conducted following the WHO insecticide susceptibility test protocol (WHO, 2016) and detailed in Balkew et al. (2021). In this study, we randomly selected 20 susceptible and 20 resistant *An. stephensi* mosquitoes from Dire Dawa post-exposure to permethrin and determined the frequency of *kdr* mutations.

## 3. Results

### 3.1. Kdr mutation population frequency

**Fig. 2.**
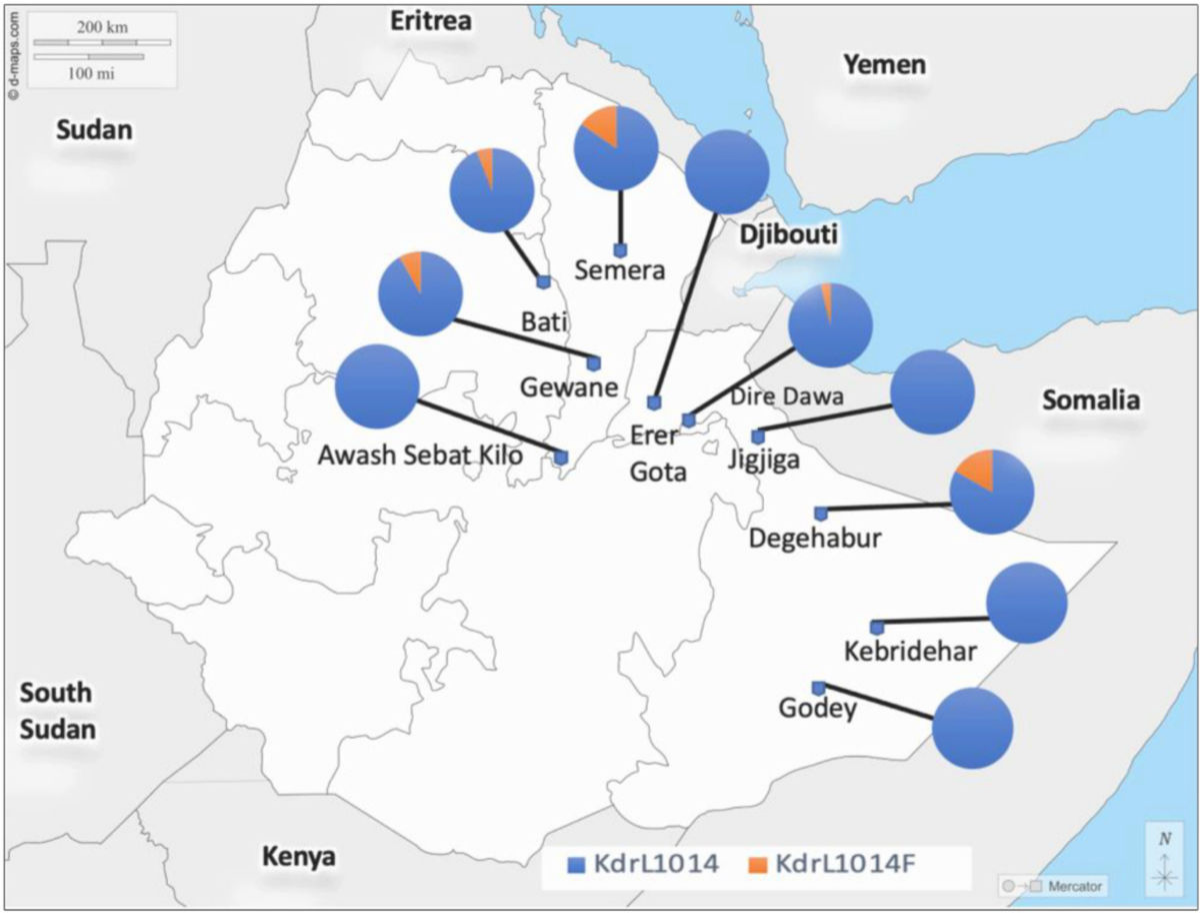
Distribution of *An. stephensi* with *kdr* mutation (*kdr*L1014F) haplotype per site in eastern Ethiopia.The blue color in the pie charts represents the wild type *kdr*L1014 haplotype and the orange color represents the mutant *kdr*L1014F haplotype.

Of the 159 *An. stephensi* specimens analyzed, nine (5.7%) carried the *kdr* mutation, particularly the *kdr* L1014F (leucine to phenylalanine). All the *kdr* alleles were heterozygous and were only observed in Semera, Bati, Gewane, Dire Dawa, and Degehabur (Fig. 2, Table 2). The *kdr* L1014S was not observed in this study.

### 3.2. Genotypic analysis across phenotype

We further randomly selected and analyzed the *vgsc* gene of 20 susceptible *An. stephensi* and 20 resistant *An. stephensi* specimens post-exposure to permethrin (i.e., survival from the bioassay tests) from Dire Dawa for the *kdr* mutation. No *kdr* mutation was detected in the permethrin-resistant sample set. However, one *kdr* L1014F mutation (heterozygous) was observed in the permethrin susceptible group (Fig. S1). Thus, no association was found between *kdr* mutation and permethrin-resistant in this population of *An. stephensi* from Dire Dawa.

### 3.3. Genetic diversity

We analyzed a 68 bp fragment of the *kdr* neighboring downstream intron and 441 bp of the upstream intron for a total of 158 individuals. We identified five polymorphic sites leading to the definition of four haplotypes in the downstream intron. The number of haplotypes in the downstream intron was similar for each site, varying between two for Degehabur to four for Awash Sebat Kilo (Table 3). The haplotype diversity (H_d_) of the downstream intron ranged from 0.344 for Degehabur to 0.722 for Dire Dawa. Overall, H_d_ was high in the northern and central populations and low in the southern population (H_d_ = 0.579, H_d_ = 0.657, and H_d_ = 0.397, respectively). Nucleotide diversity (Pi) ranged from 0.00506 to 0.02171 for Degehabur and Kebridehar, respectively. The trends in nucleotide diversity were somewhat different across subregions, with the highest nucleotide diversity observed in the central and southern regions and lowest in the north (Pi = 0.01638, 0.01531, and 0.01038, respectively) (Table 3). In the upstream intron, the number of haplotypes was eleven, varying between six for Godey to eight for Gewane, Semera, and Awash Sebat Kilo (Table 3). Haplotype diversity and nucleotide diversity had similar trends for the upstream intron. Populations from the northern and central regions had the most haplotype diversity and nucleotide diversity, while the southern population had the least haplotype diversity and nucleotide diversity (Hd = 0.850, 0.812, and 0.718; Pi = 0.00859, 0.00798, and 0.00725, respectively) (Table 3). The analysis of the combined trimmed upstream and downstream introns with the *kdr* exon also showed the northern and central populations had the highest haplotype diversity, while the southern population had the lowest value (Hd = 0.746, 0.657, 0.515, respectively) (Table 3). However, the northern and southern populations had the highest nucleotide diversity, while the lowest was observed in the central population (Pi = 0.01145, 0.01308, 0.01079, respectively) (Table 3).

### 3.4. Neutrality tests

Tajima’s D values for the *kdr* combined trimmed upstream and downstream introns with *kdr* exon were overall negative for all subregion populations except the southern population. The negative Tajima’s D values in the northern and central populations indicate an excess of low frequency polymorphism than the expectation under the neutral model of evolution. However, this deviation from neutrality was not significant (i.e., *P* > 0.05) (Table 4). Fu and Li’s D* and F* tests’ results were also overall negative for all subregion populations except the southern population, indicating an excess of rare haplotypes over what would be expected under neutrality in the northern and central populations. However, the neutral null hypothesis was not rejected following these tests (P > 0.05) (Table 4).

### 3.5. Phylogenetic analysis

**Fig. 3.**
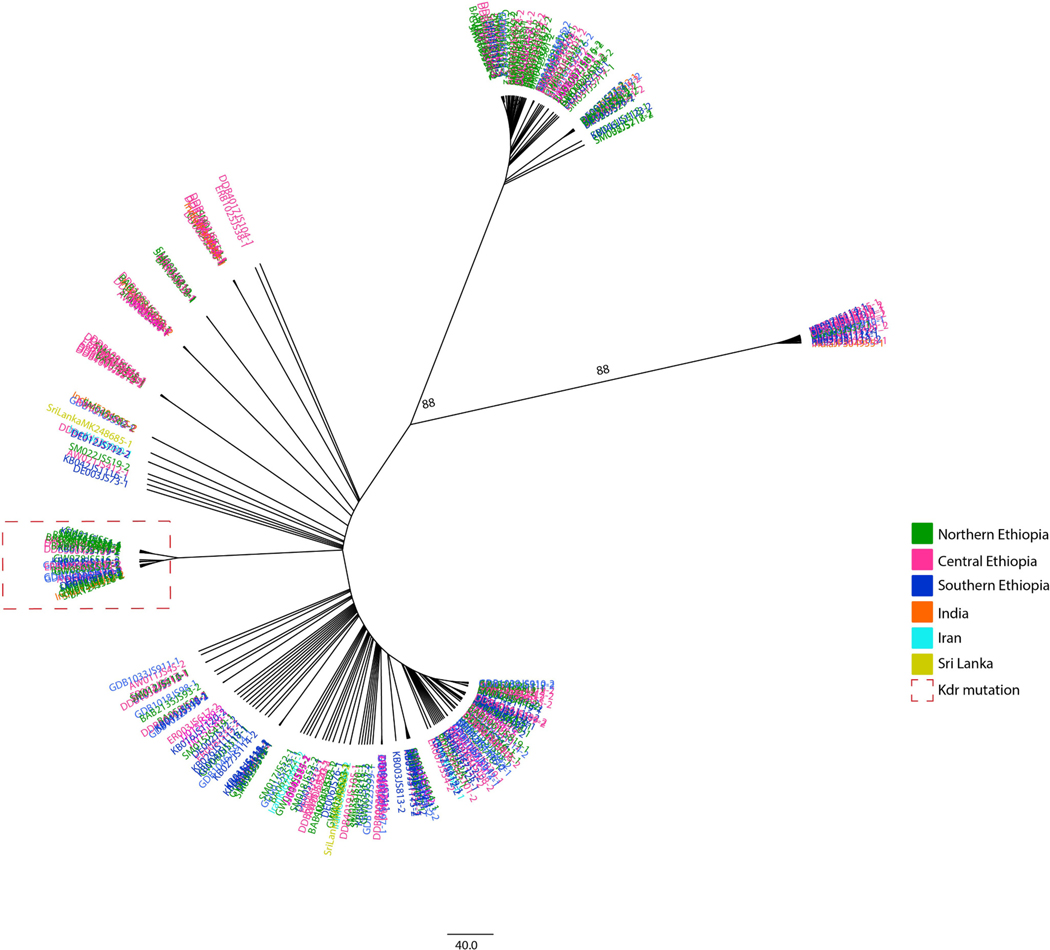
Phylogenetic tree of *kdr* exon and downstream neighboring intron in eastern Ethiopian *An. stephensi* and available global *An. stephensi*. The evolutionary history was inferred by using the Maximum Likelihood method based on the General Time Reversible model. The tree with final ML optimization likelihood (−173.70) and bootsrap values >70 are shown.

*Kdr* phylogenetic analysis included both the *kdr* exon and adjacent downstream intron segments of the *vgsc* gene from the Ethiopian samples as well as from available sequences from India, Iran, and Sri Lanka of the same *vgsc* gene region retrieved from Genbank. From these sequences, only one of the Indian sequences had the *kdr* mutation L1014F. All sequences with the *kdr* L1014F mutation fell under one clade (bootstrap = 88) (Fig. 3). The distribution of the haplotypes and bootstrap values (88 and 88) suggest some differentiation among the Ethiopian samples based on geography (i.e., northern, central, and southern) with the *kdr* mutation background intron haplotype found everywhere (Fig. 3).

### 3.6. Haplotype network analysis and population differentiation

**Fig. 4.**
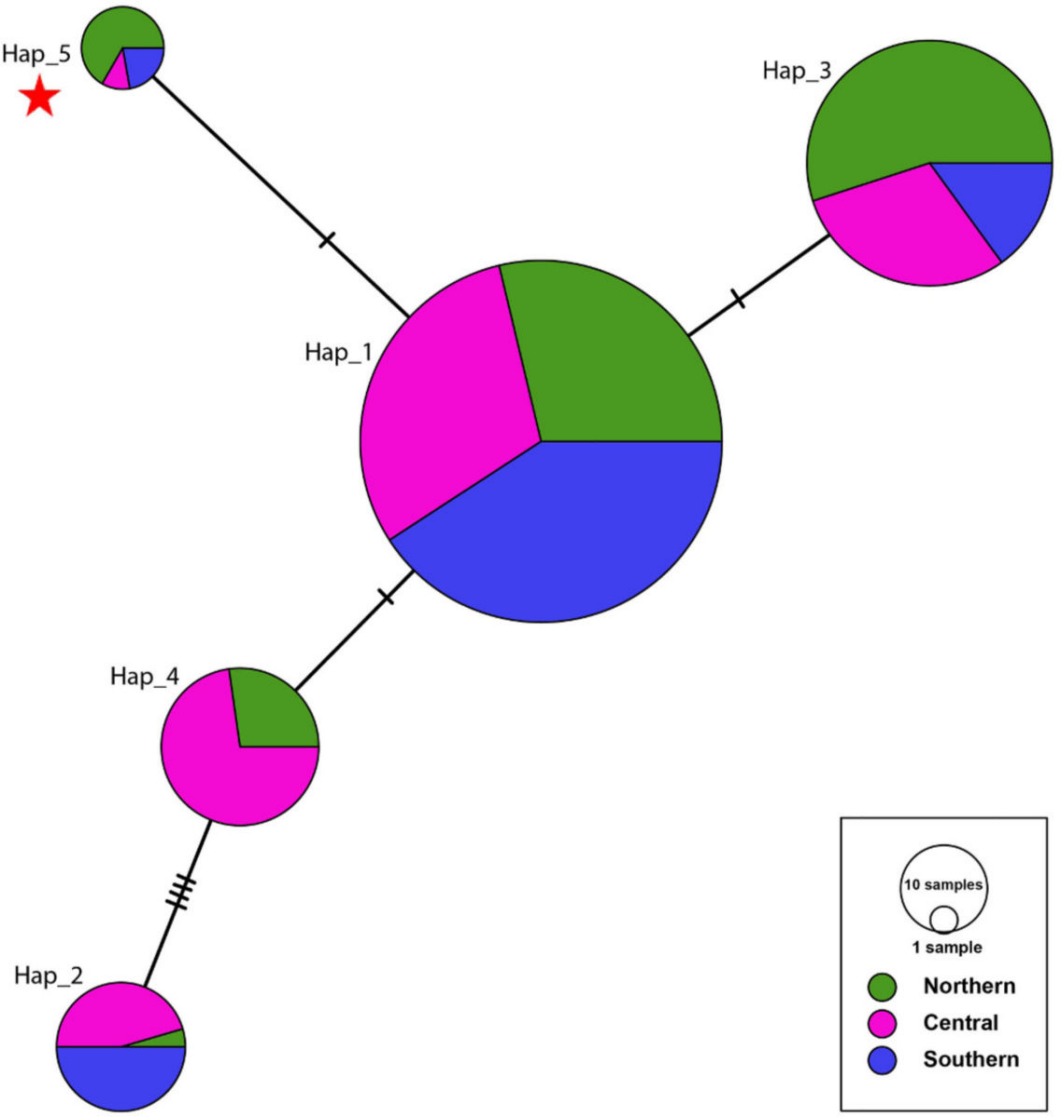
Minimum spanning network of eastern Ethiopian *An. stephensi kdr* exon and downstream *intron* haplotypes. Colors represent Ethiopian regions. Each node represents a haplotype and the proportion of that haplotype contributed by each Ethiopian region. The size of the nodes is proportional to the sample size. The ticks between nodes represent the number of nucleotide differences. The node with the red star represents the *kdr* mutation haplotype.

Similar to the phylogenetic analysis, the haplotype network analysis also reveals some differentiation among the Ethiopian samples and a widespread *kdr* haplotype background. The *kdr* L1014F mutation located on haplotype 5 is only one nucleotide different from haplotype 1, which is the most abundant haplotype (54.7%) found in all subregions of eastern Ethiopia (Fig. 4). However, haplotype 4 was only observed in the northern and central subregions making their populations more diverse than the southern population. Similarly, pairwise Fst and Φst reveal some population differentiation between the northern and southern subregion populations (Tables 5a-5b). Significant population differentiation (*p*-values <0.001) was observed between Kebridehar (southern) and all three sites of the northern subregions (Semera, Fst = 0.170, Φst = 0.179; Bati, Fst = 0.192, Φst = 0.186; and Gewane, Fst = 0.222, Φst = 0.155) (Tables 5a-5b).

## 4. Discussion

We report the presence of the *kdr* mutation in *An. stephensi* populations across eastern Ethiopia (Table 2; Fig. 2; Fig. 3). While this is the first report of *kdr* mutations in *An. stephensi* in Ethiopia, the frequency detected is low; only the *kdr* L1014F mutation was observed at a frequency of 5.7%, and all the alleles were heterozygous (1014F/1014L). *An. stephensi* with *kdr* mutations were observed in Semera, Bati, Gewane, Dire Dawa, and Degehabur. No *kdr* mutations were present in the samples from Awash Sebat Kilo, Erer Gota, Jigjiga, Kebridehar, and Godey. This finding is consistent with previous reports of *kdr* mutations in *An. stephensi* in India and Afghanistan, where the *kdr* mutation alleles were observed at a low frequency (Ahmad et al., 2016; Dykes et al., 2016); thus, suggesting that *kdr* mutations alone may not be a significant mechanism of pyrethroids resistance in *An. stephensi*.

To characterize the evolutionary history of the *kdr* mutation in *An. stephensi* in eastern Ethiopia, we conducted a phylogenetic analysis of the neighboring introns. Our study revealed that the intronic region is diverse within each site and across sites. The detection of diversity in the intronic region is consistent with previous studies on *An. stephensi* from 2016 (Carter et al., 2022). We found that all the *kdr* mutations exist on one downstream intron haplotype, suggesting that the *An. stephensi kdr* mutation evolved on a single lineage (Fig. 3). Also, the pattern of distribution of the *kdr* mutations with the highest frequency found in the northeast region where older Ethiopian *An. stephensi* populations were reported (Carter et al., 2021) may indicate that strains carrying *kdr* mutations are imported rather than evolving locally (Table 2, Fig. 2). While the *kdr* mutation is at a low frequency in the population, the background downstream intron haplotype for the *kdr* mutation is highly prevalent (54.7%, Fig. 3, Fig. 4), suggesting that at one time, the *kdr* locus underwent a hard selective sweep. The decline of the *kdr* on this background intron haplotype may result from increased fitness costs for the *kdr* in low insecticide exposure environments (Brito et al., 2013; Platt et al., 2015). The shift to less exposure to insecticides may have occurred prior to *An. stephensi* introduction to eastern Ethiopia or during its expansion across eastern Ethiopia. As a result, the wild-type allele at the *kdr* locus on the same intronic background may have become more prevalent due to lower fitness costs. Sites where the *kdr* mutation is observed should continue to be monitored throughout the year if pyrethroids are used to control *An. stephensi* or through house hold application of pyrethroids in Ethiopia and neighboring countries.

Moreover, the phylogenetic analysis of the Ethiopian *An. Stephensi kdr* exon and downstream intron sequences, including all available *An. stephensi kdr* exon and neighboring downstream Genbank sequences from other *An. stephensi* popuations (i.e., India, Iran, and Sri Lanka), revealed shared haplotypes (Fig. 3). Only the downstream intron was analyzed due to sequence availability in Genbank. The shared haplotypes confirm international connectedness and supports *kdr* mutation allele importation into Ethiopia rather than a local mutation event and subsequent local selection post introduction. However, with only three countries outside of Ethiopia with available *An. stephensi kdr* sequences, more *kdr* sequence data from Asian and African countries with *An. stephensi* are needed to specifically indicate the region of origin of *kdr* mutations observed in eastern Ethiopia.

No *kdr* mutation was detected in the permethrin-resistant *An. stephensi* from the bioassays. On the contrary, one *kdr* L1014F mutation was observed in the permethrin-susceptible sample set (Fig. S1). Moreover, metabolic resistance rather than target site insensitivity was more involved in pyrethroid resistance mechanisms in *An. stephensi* in regions where it is a major vector (Enayati et al., 2020). Furthermore, recent synergist bioassays on pyrethroid-resistant *An. stephensi* in eastern Ethiopia using piperonyl butoxide (PBO) revealed the involvement of oxidases (Balkew et al., 2021). Thus, investigations of other molecular mechanisms of pyrethroid resistance in *An. stephensi* across eastern Ethiopia are warranted.

## 5. Conclusion and future directions

The present study revealed the presence of the *kdr* mutation at a low frequency in the *An. stephensi* population across eastern Ethiopian. The *kdr* mutation is mainly observed in the northeast and cities close to the eastern border. The single background haplotype for the *kdr* mutation was also observed in some countries with established *An. stephensi*, which supports the notion that the *kdr* mutations observed in Ethiopia originated in *An. stephensi* populations prior to its introduction into Ethiopia. Investigations into the origin of *kdr* variants would benefit from additional genetic analysis from established *An. stephensi* population in and outside the Horn of Africa. These findings have significant implications on the planning and implementation of *An. Stephensi* control in Ethiopia. With the presence of the *kdr* mutation and the previous evidence of *An. stephensi*’s pattern of spread into south Ethiopia, further monitoring of *kdr* mutations and resistance phenotypes should be conducted. Additionally, further studies using genome wide multilocus estimates of gene flow will provide additional insight into predicting how emerging *kdr* mutations will spread.

Moreover, the lack of *kdr* mutations in phenotypically pyrethroid-resistant *An. stephensi* emphasizes the need to investigate other molecular mechanisms of resistance in Ethiopian *An. stephensi*. More molecular surveillance for insecticide resistance in *An. stephensi* in the Horn of Africa is warranted to understand its evolutionary history in the region, while innovative vector control strategies are also considered in controlling this invasive malaria vector.

## Supplementary Material

supplementary spreadsheet

supplementary document

## Figures and Tables

**Table 1:** List of primer and conditions used for *kdr* and neighboring introns PCR assays.

Vgsc Region	Primer	Sequence	Annealing Temperature (°C)	Final Primer Concentration (μM)

*Kdr* allele and Intron 20 (349 bp)	*Kdr*F	GGACCAYGATTTGCCAAGAT	50	0.5
	VGS_1R	CGAAATTGGACAAAAGCAAGG	50	0.5
Intron 19 (589 bp)	KdrI19_F3	GAAATTCGCTCGCACCATATCA	55	0.5
	VgscI19R	GCAACAGTCTTGCGAAAAG	55	0.5

**Table 2:** Frequency of *kdr* L1014F in eastern Ethiopian *An. stephensi.*

Site	N	*Kdr* L1014F (%)

Awash Sebat Kilo (AW)	8	0
Bati (BA)	17	1 (5.9)
Dire Dawa (DD)	26	1 (3.8)
Degehabur (DE)	12	2 (16.7)
Erer Gota (ER)	13	0
Godey (GD)	13	0
Gewane (GW)	12	1 (8.3)
Jigjiga (JJ)	9	0
Kebridehar (KB)	23	0
Semera (SM)	26	4 (15.4)
**Total**	**159**	**9 (5.7)**

**Table 3:** Genetic variation of *kdr* neighboring downstream intron (a), upstream (b) intron and combined (c) trimmed upstream and downstream with *kdr* exon in the *vgsc* of eastern Ethiopian *An. stephensi,* where n = number of genes (two per individuals), S = number of polymorphic (i.e., segregating) sites, k = average number of pairwise nucleotide differences, Pi = nucleotide diversity, h = number of haplotypes, Hd = haplotype diversity.

a.						
Downstream Intron						
Sites from Collections in eastern Ethiopia	n	S	k	Pi	h	Hd

**Northern**	**110**	**5**	**0.706**	**0.01038**	**4**	**0.579**
Bati	34	2	0.665	0.00978	3	0.590
Gewane	24	5	0.928	0.01364	4	0.601
Semera	52	2	0.650	0.00956	3	0.578
**Central**	**110**	**5**	**1.114**	**0.01638**	**4**	**0.657**
Awash Sebat Kilo	18	5	1.203	0.01769	4	0.549
Dire Dawa	48	5	1.411	0.02076	4	0.722
Erer-Gota	26	2	0.692	0.01018	3	0.600
Jigjiga	18	2	0.765	0.01125	3	0.647
**Southern**	**96**	**5**	**1.041**	**0.01531**	**3**	**0.397**
Degehabur	24	1	0.344	0.00506	2	0.344
Godey	26	5	0.631	0.00928	3	0.385
Kebridehar	46	5	1.476	0.02171	3	0.414
**All**	**316**	**5**	**0.995**	**0.01464**	**4**	**0.584**
b.						
Upstream Intron						
Sites from Collections in eastern Ethiopia	n	S	k	Pi	h	Hd

**Northern**	**102**	**14**	**3.789**	**0.00859**	**9**	**0.850**
Bati	24	12	3.696	0.00838	7	0.837
Gewane	22	13	4.268	0.00968	8	0.883
Semera	56	14	3.673	0.00833	8	0.845
**Central**	**88**	**15**	**3.520**	**0.00798**	**9**	**0.812**
Awash Sebat Kilo	12	15	4.424	0.01003	8	0.894
Dire Dawa	30	11	3.467	0.00786	5	0.789
Erer-Gota	34	12	3.344	0.00758	6	0.795
Jigjiga	12	11	3.606	0.00818	6	0.879
**Southern**	**104**	**13**	**3.197**	**0.00725**	**8**	**0.718**
Degehabur	20	11	3.489	0.00791	6	0.847
Godey	18	8	2.987	0.00677	4	0.739
Kebridehar	66	12	2.920	0.00662	7	0.632
**All**	**294**	**15**	**3.590**	**0.00814**	**11**	**0.809**
c.						
Combined Upstream and Downstream Introns with *kdr* exon						
Sites from Collections in eastern Ethiopia	n	S	k	Pi	h	Hd

**Northern**	**88**	**12**	**2.118**	**0.01145**	**10**	**0.746**
Bati	20	7	1.700	0.00919	6	0.732
Gewane	22	12	2.697	0.01458	8	0.801
Semera	46	8	2.077	0.01123	7	0.742
**Central**	**78**	**13**	**1.996**	**0.01079**	**9**	**0.657**
Awash Sebat Kilo	12	12	2.682	0.01450	6	0.682
Dire Dawa	30	6	2.149	0.01162	4	0.692
Erer Gota	24	6	1.804	0.00975	4	0.612
Jigjiga	12	6	1.515	0.00819	5	0.667
**Southern**	**70**	**11**	**2.421**	**0.01308**	**5**	**0.515**
Degehabur	20	6	1.400	0.00757	4	0.553
Godey	18	9	1.627	0.00880	3	0.503
Kebridehar	32	9	3.046	0.01647	3	0.462
**All**	**236**	**13**	**2.214**	**0.01197**	**12**	**0.664**

**Table 4:** Combined *kdr* neighboring trimmed upstream and downstream introns with *kdr* exon Neutrality Tests.

Sites from Collections in eastern Ethiopia	n	Tajima’s D Test Estimate (p-value)	Fu & Li’s D* Test Estimate (p-value)	Fu & Li’s F* Test Estimate (p-value)

**Northern**	**88**	**−0.29493** (**P > 0.10)**	**−0.97666 (P > 0.10)**	**−0.87490 (P > 0.10)**
Bati	20	−0.45507 (P > 0.10)	0.67395 (*P* > 0.10)	0.40934 (*P* > 0.10)
Gewane	22	−0.63319 (*P* > 0.10)	−0.65800 (*P* > 0.10)	0.75667 (P > 0.10)
Semera	22	−0.63319 (P > 0.10)	−0.65800 (P > 0.10)	−0.75667 (P > 0.10)
**Central**	**78**	**−0.68015 (P > 0.10)**	**1.88463 (P > 0.10)**	**−1.73539 (P > 0.10)**
Awash Sebat Kilo	12	−1.36573 (P > 0.10)	−0.90334 (P > 0.10)	−1.16288 (P > 0.10)
Dire Dawa	30	1.19627 (P > 0.10)	0.43925 (P > 0.10)	0.77473 (P > 0.10)
Erer Gota	24	0.37197 (P > 0.10)	0.49737 (P > 0.10)	0.53477 (P > 0.10)
Jigjiga	12	−0.90540 (P > 0.10)	−0.50357 (P > 0.10)	−0.68730 (P > 0.10)
**Southern**	**70**	**0.16620 (P > 0.10)**	**0.81478 (P > 0.10)**	**0.70176 (P > 0.10)**
Degehabur	20	−0.54876 (P > 0.10)	0.54727 (P > 0.10)	0.27570 (P > 0.10)
Godey	18	−1.33960 (P > 0.10)	−2.11077 (0.10 > *P* > 0.05)	−2.18628 (0.10 > P > 0.05)
Kebridehar	32	1.11241 (P > 0.10)	0.18169 (P > 0.10)	0.54838 (P > 0.10)
**All**	**236**	**0.06949 (P > 0.10)**	**0.79098 (P > 0.10)**	**0.62317 (P > 0.10)**

**Table 5a:** Population Pairwise Fst based on haplotype frequencies. Fst values are plotted below the diagonal and *p*-values on the top. Color gradient based on Fst values (yellow = highest, orange = lowest).

		Northern			Central				Southern		

		Semera	Bati	Gewane	Awash	Erer-Gota	Dire Dawa	Jigjiga	Degehabur	Kebridehar	Godey
Northern	**Semera**	.	0.88288	0.66667	0.08108	0.1982	0.01802	0.85586	0.10811	<0.001	0.01802
	**Bati**	−0.02119	.	0.89189	0.09009	0.06306	0.03604	0.8018	0.05405	<0.001	0.01802
	**Gewane**	−0.01793	−0.02907	.	0.07207	0.07207	0.04505	0.47748	0.05405	<0.001	0.00901
Central	**Awash Sebat Kilo**	0.05228	0.06449	0.09997	.	0.4955	0.03604	0.31532	0.56757	0.27928	0.54054
	**Erer-Gota**	0.02365	0.03111	0.06516	−0.02316	.	0.11712	0.64865	0.13514	<0.001	0.0991
	**Dire Dawa**	0.04533	0.05234	0.05462	0.06296	0.02999	.	0.28829	<0.001	<0.001	<0.001
	**Jigjiga**	−0.02784	−0.03233	−0.01641	0.0122	−0.02338	0.01432	.	0.22523	<0.001	0.10811	
Southern	**Degehabur**	0.0471	0.05842	0.08808	−0.01786	0.03136	0.123	0.03623	.	0.03604	0.75676
	**Kebridehar**	0.16997	0.19221	0.22209	0.01313	0.11114	0.15763	0.15807	0.05983	.	0.13514
	**Godey**	0.08806	0.09788	0.13509	−0.01951	0.05185	0.14986	0.0714	−0.02613	0.03679	.

**Table 5b:** Pairwise Φst between study sites based on genetic distance. Φst values are plotted below the diagonal and p-values on the top. Color gradient based on Φst values (yellow = highest, orange = lowest).

		Northern			Central				Southern		

		Semera	Bati	Gewane	Awash	Erer-Gota	Dire Dawa	Jigjiga	Degehabur	Kebridehar	Godey
Northern	**Semera**	.	0.85586	0.54955	0.04505	0.13514	<0.001	0.73874	0.1982	<0.001	0.12613
	**Bati**	−0.01884	.	0.9009	0.03604	0.05405	<0.001	0.66667	0.08108	<0.001	0.07207
	**Gewane**	−0.01292	−0.02729	.	0.07207	0.0991	0.00901	0.41441	0.07207	<0.001	0.07207
Central	**Awash**	0.05705	0.07976	0.07014	.	0.68468	0.20721	0.36036	0.17117	0.28829	0.59459
	**Sebat Kilo**										
	**Erer-Gota**	0.04039	0.05989	0.07204	−0.0281	.	0.04505	0.55856	0.07207	0.04505	0.24324
	**Dire Dawa**	0.1353	0.13446	0.107	0.0225	0.05844	.	0.01802	<0.001	0.10811	0.01802
	**Jigjiga**	−0.02276	−0.02525	−0.01631	0.00677	−0.01395	0.07072	.	0.27027	0.00901	0.28829
Southern	**Degehabur**	0.01744	0.04461	0.0559	0.03677	0.05694	0.14508	0.03224	.	0.01802	0.63063
	**Kebridehar**	0.17915	0.18643	0.15538	0.0169	0.09672	0.02615	0.1225	0.14001	.	0.02703
	**Godey**	0.02918	0.0457	0.04911	−0.01237	0.02358	0.10551	0.01235	−0.01734	0.0891	.

## Data Availability

All data generated or analyzed during this study are included in this published article and its supplemental information files.

## Abbreviations:

BLAST: Basic Local Alignment Search Tool
CDC: Centers for Disease Control and Prevention
DNA: deoxyribonucleic acid
IRS: Indoor Residual Spraying
ITNs: Insecticide Treated Nets
kdr: knockdown resistance
LLINs: Long Lasting Insecticide Nets
NCBI: National Center for Biotechnology Information
PCR: polymerase chain reaction
PSC: pyrethrum spray catch
vgsc: voltage-gated sodium channel
WHO: World Health Organization
