# Supplementary material for: Detection and population genetic analysis of kdr L1014F variant in eastern Ethiopian Anopheles stephensi
Source: Infect Genet Evol. Author manuscript; Available in PMC 2025 Apr 1. (PMC11959535; doi:10.1016/j.meegid.2022.105235)
Supplement: supplementary document [file NIHMS1977036-supplement-supplementary_document.docx]

**Detection and population genetic analysis of *kdr* L1014F variant in eastern Ethiopian *Anopheles stephensi***

**Supplemental material**

**Figure S1.** Frequency of *kdr* L1014 genotypes in permethrin-susceptible versus permethrin-resistant *An. stephensi* post-bioassay from Dire Dawa, Ethiopia.
